# The maternal and newborn health eCohort to track longitudinal care quality: study protocol and survey development

**DOI:** 10.1080/16549716.2024.2392352

**Published:** 2024-08-20

**Authors:** Catherine Arsenault, Katherine Wright, Tefera Taddele, Ashenif Tadele, Anagaw Derseh Mebratie, Firew Tiruneh Tiyare, Rose J. Kosgei, Jacinta Nzinga, Bethany Holt, Irene Mugenya, Emma Clarke-Deelder, Adiam Nega, Dorairaj Prabhakaran, Sailesh Mohan, Nompumelelo Gloria Mfeka-Nkabinde, Londiwe Mthethwa, Damen Haile Mariam, Gebeyaw Molla, Theodros Getachew, Prashant Jarhyan, Monica Chaudhry, Munir Kassa, Margaret E. Kruk

**Affiliations:** a Department of Global Health, Milken Institute School of Public Health, George Washington University, Washington, DC, USA; b Department of Global Health and Population, Harvard T.H. Chan School of Public Health, Boston, MA, USA; c Health System Research Directorate, Ethiopian Public Health Institute, Addis Ababa, Ethiopia; d School of Public Health, College of Health Sciences, Addis Ababa University, Addis Ababa, Ethiopia; e Department of Obstetrics and Gynecology, University of Nairobi, Nairobi, Kenya; f Health Economics Research Unit, KEMRI Wellcome Trust Research Program, Nairobi, Kenya; g Blavatnik Institute of Global Health and Social Medicine, Harvard Medical School, Boston, MA, USA; h Department of Epidemiology and Public Health, Swiss Tropical & Public Health Institute, Allschwil, Switzerland; i Public Health Foundation of India, Gurgaon, India; j Centre for Chronic Disease Control, New Delhi, India; k School of Nursing and Public Health, University of KwaZulu-Natal, Durban, South Africa; l Public Health Foundation of India, Gurugram, Haryana, India; m Ministry of Health of Ethiopia, Addis Ababa, Ethiopia

**Keywords:** Health system quality, maternal and newborn health, implementation science, evidence-based care, quality of care

## Abstract

The MNH eCohort was developed to fill gaps in maternal and newborn health (MNH) care quality measurement. In this paper, we describe the survey development process, recruitment strategy, data collection procedures, survey content and plans for analysis of the data generated by the study. We also compare the survey content to that of existing multi-country tools on MNH care quality. The eCohort is a longitudinal mixed-mode (in-person and phone) survey that will recruit women in health facilities at their first antenatal care (ANC) visit. Women will be followed via phone survey until 10-12 weeks postpartum. User-reported information will be complemented with data from physical health assessments at baseline and endline, extraction from MNH cards, and a brief facility survey. The final MNH eCohort instrument is centered around six key domains of high-quality health systems including competent care (content of ANC, delivery, and postnatal care for the mother and newborn), competent systems (prevention and detection, timely care, continuity, integration), user experience, health outcomes, confidence in the health system, and economic outcomes. The eCohort combines the maternal and newborn experience and, due to its longitudinal nature, will allow for quality assessment according to specific risks that evolve throughout the pregnancy and postpartum period. Detailed information on medical and obstetric history and current health status of respondents and newborns will allow us to determine whether women and newborns at risk are receiving needed care. The MNH eCohort will answer novel questions to guide health system improvements and to fill data gaps in implementing countries.

## Paper context

- **Added knowledge:** The MNH eCohort will answer novel questions and provide information on undermeasured dimensions of MNH care quality included continuity of care, system competence, and user experience.
- **Global health impact for policy and action:** The data generated will inform policy makers to develop strategies to improve adherence to standards of care and quality for mothers and newborns.

## Background

Despite substantial progress in the past decades, about 287,000 women die during pregnancy or childbirth and 2.4 million newborns die within the first 28 days of life [1]. More than 95% of these deaths occur in low- and middle-income countries (LMICs) [1]. It is also estimated that 50%-61% of these deaths are among women and newborns who used the health system but received poor quality services [2]. Maternal and newborn health (MNH) morbidity and mortality are regarded as sensitive indicators of how well a health system is functioning because a large share of the burden can be averted by high-quality health systems.

The Lancet Global Health Commission on High Quality Health Systems in the Sustainable Development Goals (SDG) era (the HQSS Commission) proposed a new framework to assess health system quality centered around the processes of care and outcomes that matter most to people and to good health [3]. The HQSS Commission compiled data from a wide range of countries and sources to describe the state of quality in LMICs [3]. Review of available data revealed that most health system measurement efforts in LMICs tend to focus on the structural quality of health facilities and on the level of health service utilization. For example, the Service Provision Assessment (SPA) surveys and the Service Availability and Readiness Assessment (SARA) survey are primarily focused on structural quality [4]. Measuring the availability of tools, equipment, workforce, and platforms for care, is crucial to health system functioning but these items do not by themselves guarantee high-quality care and better health [5]. The Demographic and Health Surveys (DHS) and Unicef’s Mutiple Indicators Cluster Surveys (MICS) are primarily focused on measuring levels of health service utilization. Utilization rates for antenatal care (ANC) or facility-based delivery care provide little information on the quality of services provided and are not always linked to improved outcomes. The HQSS Commission revealed that little data exists on competent care and systems, user experience, and on the links between quality care and health, confidence in the health system, and economic outcomes [3].

In addition, evidence on the quality of MNH care in LMICs has largely been based on cross-sectional data which are limited for quality assessments and fail to capture the dynamic elements of MNH care from first ANC visit through delivery and postnatal care (PNC) [6]. Understanding how process quality is linked to outcomes over time, across the MNH continuum, is crucial to develop evidence-informed policies. The maternal and newborn health eCohort was designed to address these gaps in health system quality measurement in LMICs. The MNH eCohort takes advantage of the increase in mobile phone connectivity and ownership in LMICs and aims to provide a flexible measurement tool to assess care quality across the patient journey.

The eCohort is focused on the user and combines several patient-reported outcomes and experience measures (PROMS and PREMS). The eCohort’s focus on PROMS and PREMS constitutes a novel approach to assessing quality in LMIC contexts. PROMS and PREMS are increasingly integrated in electronic health records (EHRs) in high-income countries and their measurement is recognized as a priority for delivering high-value care [7]. The eCohort will contribute to improvements in health system monitoring in low resource settings where EHRs and web-based patient portals are currently rarely available.

In this paper, we describe the MNH eCohort methodology, the survey development process, the survey content and sections, and the future research and policy use of the data generated by the MNH eCohorts. We also compare the survey to existing, multi-country tools, that have aimed to measure MNH quality.

## Methods and analysis

### Aim and study design

The MNH eCohort is a longitudinal mixed mode (in-person and phone) survey aimed at collecting near-real time data on health system quality in selected sites. The MNH eCohort has four goals: (1) to measure system competence across the continuum of MNH care, (2) to describe health outcomes, user experience, and care pathways in the health system for women and newborns, (3) to identify gaps in effective care provision for good health outcomes, and (4) to build a flexible measurement tool for assessment of health system performance using mobile phones.

### Survey development process

The MNH eCohort survey was developed collaboratively by members of the Quality Evidence for health System Transformation (QuEST) network based in Ethiopia, India, Kenya, South Africa, and the United States. The QuEST network, created in response to the findings of the HQSS Commission, is an initiative focused on the measurement and improvement of health system quality through multi-country partnerships. Members of the development group included health system researchers, policy makers, obstetricians, medical professionals as well as survey designs experts. Prior to designing the survey, a review of existing multi-country surveys covering MNH quality was conducted, and dimensions of quality identified in these surveys were mapped onto the HQSS Commission framework. Members of the development group identified gaps in the measurement of care processes (competent care and systems and user experience) and outcomes (health, confidence in the health system and economic benefits).

The MNH eCohort tool was therefore centered around these domains. The development group met regularly to review study goals, instrument sections, and to ensure content validity and relevance to local contexts. Content validity was further tested through external peer review by health system experts and survey methods specialists. To ensure the survey was locally applicable and interpretable, the questionnaire was assessed for comprehension in each country via cognitive interviews. The cognitive interviews were conducted among pregnant women in study countries and focused on ensuring respondents were able to comprehend and interpret the survey questions, they were able to answer the questions confidently and were able to recall the specific things that occurred during the health care visits. The instrument was translated into local languages by professional translators and pre-tests were conducted to refine question wording and local response options. Corrections were made by local research teams prior to mainstage data collection.

The MNH eCohort instrument combines existing and previously validated survey questions from past studies as well as newly developed items. For example, the eCohort borrowed survey questions from the DHS, MICS, the Performance Monitoring for Action (PMA) study, the Commonwealth Fund International Health Policy survey, the WHO STEPwise approach to NCD risk factor surveillance (STEPS) survey, the World Health Survey, the World Mental Health Survey, the Ethiopian National Health Accounts survey, and various maternal and newborn health studies previously conducted by members of the development group [8–19]. To identify elements of competent care across the MNH continuum, the development group used World Health Organization (WHO) and national guidelines on ANC, delivery and PNC. In addition to local guidelines from the four implementing countries, this included the WHO 2016 recommendations on ANC for a positive pregnancy experience, the WHO 2018 recommendations on intrapartum care, the WHO 2022 recommendations on maternal and newborn care for a positive postnatal experience, and the WHO 2020 guideline on improving early childhood development [20–23].

The survey instrument is centered around the six key domains of the HQSS Commission framework: competent systems, competent care, user experience, health outcomes, confidence in the health system, and economic outcomes [3]. The following section describes the key dimensions of quality assessed in the survey.

#### Competent systems

A key aspect of high-quality health systems relates to whether people receive safe, timely, continuous, and integrated care for all health needs. High quality health systems should also prevent and detect health problems in a timely manner. The eCohort will provide evidence on the following elements of competent health systems.

##### Prevention and timely detection

During pregnancy, childbirth and in the newborn and postpartum periods, health providers should detect, communicate, and treat any existing and emerging health problems. At baseline, the MNH eCohort will assess whether pregnant women have been previously diagnosed with common chronic conditions (hypertension, diabetes, HIV, mental health, heart disease), whether they have a history of obstetric complications (e.g. prior stillbirths, neonatal deaths, caesarean sections, or postpartum hemorrhages), and whether the current pregnancy involves certain risk factors (e.g. age, gravidity, multiple gestation, undernutrition, anemia, or high blood pressure).

Women will also be asked whether they have discussed these conditions, risk factors, or previous obstetric complications with their provider and whether they have received any advice, treatment or referral. This will allow us to estimate whether women are appropriately classified according to their needs for basic versus specialized care in pregnancy and whether risk stratification is taking place. We will assess whether women with risk factors are receiving additional or specialized care during pregnancy or delivery (such as additional visits, diagnostic tests or treatments, or visits at the hospital or with an obstetrician). The same will also be assessed for newborns in need of specialized care in the first 10–12 weeks of life.

Throughout the eCohort, women will also be asked to report any emerging health problems or danger signs for themselves (e.g. severe headaches, fever, vaginal bleeding, experience of intimate partner violence) and the newborn (e.g. trouble feeding, jaundice, diarrhea, pneumonia) and the types of advice, referral or treatment received.

##### Care continuity and integration

We will assess care continuity and integration from the user’s perspective. We define integrated care as care that is coordinated across the MNH continuum and across health visits. Care continuity and integration will be reflected by the ability of the health system to coordinate services (tests, consultations, or procedures), ensure information continuity, and manage smooth transitions from one facility to the other. For example, we will assess whether women are referred for tests when the facility cannot perform them (e.g. blood tests or ultrasounds), whether women or newborns at risk are referred for follow-up care with specialists, and whether delivery care providers have information about women’s medical history or look at women’s ANC records during childbirth. Follow-up on test results and referrals is another important component of continuous and integrated care. Women will be asked whether they have received and are aware of their test results, and similarly, whether they are following up on referrals and recommendations from their providers. The goal is to identify specific bottle necks in care continuity.

We will also describe the number of different health care facilities used across the MNH continuum giving some indication of care continuity and integration [3]. During each follow-up survey, women will report whether they had new health care consultations, the type and name of the facilities visited (or whether care was received at home through outreach programs), the purpose of the consultations for themselves and the newborn, and the reasons for using specific health facilities. This will allow us to understand the reasons for using specific facility types, and to determine, for example, whether women use the private sector or traditional birth attendants to complement public health center care.

#### Competent care

Another important dimension of high-quality health systems relates to whether care is evidence-based and whether women and newborns consistently receive recommended services across the continuum of care. Competent care includes systematic assessments (through appropriate history questions, examinations, and tests) and the provision of appropriate treatments and counseling. The MNH eCohort will assess whether these items are consistently provided across the prenatal, intrapartum, and postnatal periods (Table 1). Because the survey is largely based on self-reported information, specific care items will be limited to those that can be generally well-reported by women [24]. Given poor recall in the intrapartum period, for example, measures of the content of delivery care will be limited. For women with a maternal health card at endline, we will aim to supplement the self-reported information with data extracted from the MNH cards [25].

**Table 1.** Examples of competent care items measured in the MNH eCohort across the continuum of care.

**Prenatal care period**	
• Hemoglobin testing	• Tetanus toxoid vaccination
• Diabetes, hypertension, and depression screening	• Calcium supplementation*
• Nutritional status screening	• Malaria IPTp*
• HIV, Syphilis and Hepatitis B testing	• Treatment of common chronic conditions (depression, hypertension, diabetes, HIV, heart disease)
• TB screening*	• Support for intimate partner violence
• CD4 count and viral load testing*	• Counseling on healthy eating and exercise, smoking cessation, birth plan, and signs of pregnancy complications
• Blood and urine test	
• Ultrasound	
• Fetal heart rate monitoring	
• Food supplementation in malnourished women	
• Iron and folic acid supplementation	
• Anthelminthic treatment	
**Intrapartum period**	
• Blood pressure monitoring	• Neonatal BCG vaccination
• HIV testing	• Appropriate management of babies born to HIV+ women
• Intravenous fluids*	• Counseling on appropriate newborn care including heat, feeding, umbilical cord, washing, the importance of immunization, and newborn and maternal danger signs
• Caesarean section	
• Blood transfusion*	
• Episiotomy*	
• Provision of uterotonic drugs	
• Checkup of mother and baby before discharge	
• Baby placed skin-to-skin	
• Immediate breastfeeding initiation	
**Postnatal and postpartum care period**	
• Hypertension and depression screening	• Routine childhood immunizations
• Care of caesarean section scar	• Management of postpartum depression
• HIV testing	• Support for intimate partner violence
• Assessment of the newborn for danger signs (e.g., temperature, weight, breathing, feeding, jaundice)	• Counseling on breastfeeding, appropriate breast care, appropriate newborn care, newborn danger signs, and postpartum family planning options
• Newborn eyes and hearing screening	
• Blood and urine tests	

*Where indicated. IPTp is Intermittent Preventive Treatment of pregnancy.

Unlike previous surveys, the MNH eCohort will determine not only whether each content of care item is done at least once over the course of care but also, how many times it is done and when. For example, according to WHO guidelines, a blood test for anemia screening should be done three times in pregnancy, at 12, 26 and 36 weeks of gestation [20]. Similarly, at least four PNC visits should take place within 24 h after birth, between 48 and 72 h, between 7 and 14 days, and during week six after birth [22]. Women will be contacted by phone every month to assess care seeking and content of care, from first ANC visit to 10–12 weeks postpartum. Unlike previous surveys that assessed the content of care cross-sectionally and determined whether each item had been done at least once during the woman’s pregnancy, the eCohort should provide more precise estimates of the amount and timeliness of care. The longitudinal nature of the eCohort will also allow us to assess care competence according to specific risks that evolve throughout the pregnancy and postpartum period.

#### User experience

In addition to its intrinsic value, a positive user (or patient) experience is crucial to improve retention and confidence in health systems [3]. The HQSS Commission defined a positive user experience as care that is respectful, patient-centered, and easy to navigate. Yet, little data exists on these important dimensions of quality. The importance of patient-reported experience measures (PREMS) to assess the quality of maternity care is increasingly recognized [26].

The eCohort will survey women on their experiences with each ANC visit, with the delivery, and with PNC visits (Table 2). Women will rate different aspects of care including overall quality, the level of respect from providers, the clarity of explanations received, wait times and time spent with health providers, and whether they have experienced discrimination.

**Table 2.** Examples of user experience items measured in the MNH eCohort across the continuum of care.

**Prenatal care period**
**First ANC visit**: Overall quality rating of the first visit Wait time Time spent with the health provider User rating of ANC provider knowledge, equipment availability, provider respect, clarity of explanations, amount of time spent with provider, wait time, courtesy of facility staff
**Subsequent ANC visits**: Overall quality rating of subsequent ANC visits
**Intrapartum period**
Overall quality rating of delivery care User rating of delivery provider knowledge, equipment availability, provider respect, clarity of explanations, amount of time spent with provider, wait time, courtesy of facility staff, confidentiality, privacy, affordability Mistreatment during childbirth Consent and privacy for vaginal exams Appropriate pain relief
**Postnatal and postpartum care period**
Overall quality rating of each PNC visit Experience of discrimination during the course of care

Mistreatment of women during childbirth is well documented [27]. The eCohort includes validated survey measures on mistreatment from the WHO multi-country study: ‘How women are treated during facility-based childbirth’ [28]. We will use brief item sets that have been shown to efficiently capture two domains of mistreatment: physical abuse and verbal abuse [27,28]. Women will also rate the level of supportive environment during labor, receipt of appropriate pain relief, as well as consent and privacy during vaginal exams.

#### Health outcomes

Beyond survival (maternal death, stillbirth, fetal death, and neonatal death), the eCohort will also measure maternal and newborn morbidity and self-reported health and wellbeing. The selection of patient-reported outcome measures (PROMS) in the eCohort was guided by the recommended set of standardized outcome measures for pregnancy and childbirth developed by the International Consortium for Health Outcomes Measurement (ICHOM) [29].

Measures of maternal morbidity in the intrapartum period will include maternal need for intensive care, length of stay, c-section. episiotomy, experience of self-reported severe health problems and receipt of blood transfusion. Other PROMS will include self-rated health, health-related quality of life (HRQoL) (assessed via the EuroQol five-dimension scale questionnaire (EQ5D)), depression (assessed via the Patient Health Questionnaire-9 (PHQ-9)), obstetric fistula symptoms, pain with intercourse, confidence in and problems with breastfeeding and mother-infant attachment (tracked via the mother-to-infant bonding scale (MIBS)). The EQ5D measures five dimensions of health (mobility, self-care, usual activities, pain and discomfort and depression and anxiety) [30]. The PHQ9 is a 9-item instrument for measuring the severity of depression and has demonstrated good diagnostic operating characteristics to screen for perinatal depression [31]. The MIBS is a measure of mother-infant attachment closely linked to mood and depression [32].

For the baby, women will be asked to report on any complications experienced in the first day of life, whether they were sent to intensive care, and their length of stay at the facility after birth. For the newborn, the mother will also report self-rated health and HRQoL will be assessed through the Infant Quality of life Instrument (IQI). The IQI assesses HRQoL in 0 to 1-year-old infants through seven attributes: sleeping, feeding, breathing, stooling, mood, skin, and interaction [33].

PROMS can be used to assess the quality of care and provide new information on the impact that care has on self-assessed health and health-related quality of life [34]. PROMS are generally well correlated with health outcomes measured in clinical settings and recently, the US Food and Drug Administration (FDA) has endorsed patient-reported health status as a viable outcome for chronic disease therapy approval [35].

Finally, health measurements at baseline and endline (described further) will provide information on the nutritional status of mother and baby, anemia, high blood pressure and child growth.

#### Confidence in the health system

A summative measure of health system quality relates to the degree to which people trust their health system. Four measures of health system confidence will be administered at both baseline and endline to assess whether women’s trust in health systems has changed over time. At baseline and endline, women will be asked:

1. To rate the overall quality of medical care in their country (from poor to excellent)
2. To select one of three statement that best describes their view of the health care system in their country (1. On the whole, the system works pretty well, and only minor changes are necessary to make it work better. 2. There are some good things in our health care system, but major changes are needed to make it work better. 3. Our health care system has so much wrong with it that we need to completely rebuild it.)
3. To rate their confidence in being able to receive good quality medical care if they became very sick.
4. To rate their confidence in being able to afford good quality medical care if they became very sick without suffering financial hardship.

These survey questions were extracted from the Commonwealth Fund International Health Policy Survey and the People’s Voice Survey [11,36]. For the first ANC visit and for childbirth, women will also be asked whether they would recommend the same health facility to family and friends.

#### Economic outcomes

Finally, high quality health systems should provide care that is affordable and should protect people from catastrophic or impoverishing expenditures when seeking care, particularly for emergencies [37,38]. The eCohort will collect data on all health expenditures incurred by users for the first ANC visit, subsequent ANC visits, for labor and delivery care and for any PNC visits. Respondents will be asked to report any amount spent on registration costs, lab and diagnostic tests, medicines, transport and food or accommodation. Women will also report the financial sources used to pay for care such as current income, health insurance plan, sale of assets, or borrowing.

### Sample and respondent selection

Two sentinel sites will be selected in each implementing country (one predominantly rural, and one predominantly urban). Our target population will be pregnant women who use ANC services in each site (e.g. a zone in Ethiopia, county in Kenya, district municipality in South Africa and a district in India). Recruitment will be conducted in person at health facilities while women attend their first ANC consultation. Women will be recruited in health facility types that are representative of care seeking patterns in the site. For example, we will use data from the health management information system (such as the DHIS2 platform) to determine the proportion of women who attend ANC in public primary care facilities, public hospitals and private clinics or hospitals. DHIS2 contains data on the total number of ANC visits by the facility and may not include the number of first visits. They may also contain errors or missing information. We will triangulate these data with those from the DHS where women report the facility types used for ANC. The facilities selected for enrollment will be selected within these four strata by probability proportional to size (using ANC patient volume as a measure of size) and with the aim to finish enrollment within two months. We aim to recruit a minimum of 50 women per facility strata in each site. There will be no restriction on the gestational age at enrollment (some women may seek ANC in their first trimester, while others may be enrolled late in their third trimester). Other inclusion criteria include being aged at least 15 years old, being at the facility to receive the first ANC visit and planning to continue to reside in the study site. We aim for 500 pregnant women to be recruited in each site (1000 per country). The sample size justification is included in supplemental materials.

### Data collection procedures

**Figure 1.**
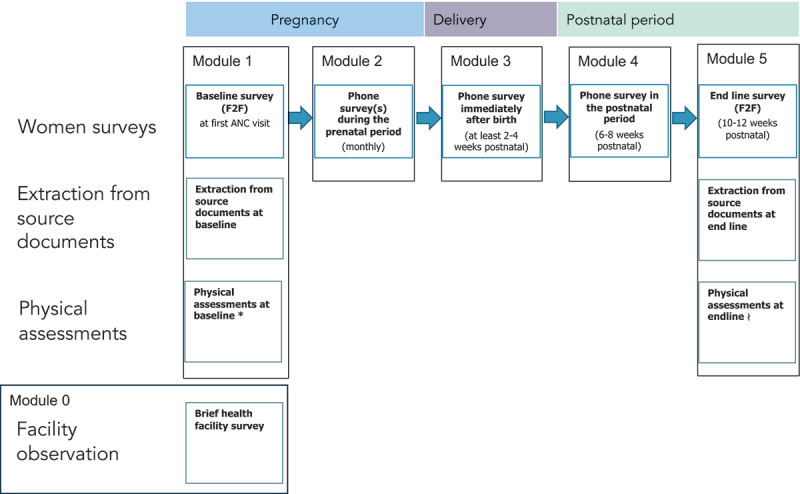
Structure of the maternal and newborn health eCohort survey.

The eCohort consists of five women survey modules (Figure 1). Module 1, the baseline survey, will be administered in person at enrollment in the health facility where women receive their first ANC visit. The baseline survey will be administered using tablets and we aim for enrollment to take no longer than 2 months. Data collectors will then contact women by phone every month (through computer-assisted-telephone interviewing) to administer the prenatal phone survey module (module 2). During these monthly calls, the data collector will determine whether women are still pregnant and whether they have given birth. Once she has delivered (or lost the pregnancy), module 3 will be administered to collect information on delivery care and outcomes. For consistency across respondents, the third module will be administered 2 to 4 weeks after the delivery. One month later, a phone survey on PNC care and outcomes will be administered (module 4, 6–8 weeks postpartum). Finally, an endline in-person survey will be conducted at 10–12 weeks postpartum at the health facility where women were enrolled. Given the sensitive nature of pregnancy losses, women who face a miscarriage will end participation in the eCohort after module 2. Those who have a stillbirth or a newborn death will complete module 3 to provide information on delivery care and end their participation. Similarly, women reporting a neonatal or infant death at module 4, will not complete the end line module.

F2F is face-to-face survey ANC is antenatal care.

*Physical assessments of the woman at baseline include blood pressure measurement, weight, height, mid-upper arm circumference measurement, hemoglobin level test to assess anemia and depression screening using the Patient Health Questionnaire-9 (PHQ9) [8].

∤Physical assessments of the mother at the endline will include blood pressure measurement, weight, height, mid-upper arm circumference measurement, hemoglobin level test to assess anemia, and depression screening using the PHQ9 [8]. Physical assessments of the baby will include weight, length, and head circumference measurement.

Follow-up time will vary depending on the stage of pregnancy at baseline. We expect the total follow-up time, from enrollment to endline, to range from three to 11 months (supplemental materials). During the baseline survey, data collectors will determine the preferred phone number to use for follow-up and they will collect a list of backup phone numbers to facilitate call tracing (e.g. phone numbers of family members or friends). The in-person surveys (baseline and endline) will take no more than 30 minutes to complete while we aim for each phone follow-up survey to last no longer than 20 minutes. The delivery care survey (module 3) may be split into two phone calls to reduce the burden on respondents.

The MNH eCohort is primarily based on self-reports, but we aim to complement self-reported information with data from three additional sources: a brief health facility survey, physical health assessments, and extraction of information from MNH cards (Figure 1). A short health facility assessment (module 0) will allow us to describe the structural quality and size of health facilities where women are recruited. The baseline and endline in-person surveys will also include brief physical assessments (women’s weight, height, blood pressure, mid-upper arm circumference, hemoglobin level and baby’s weight, length, and head circumference). The data collectors will be trained in performing these health assessments. Hemoglobin measurement (using a point-of-care test kit) will require careful training especially for data collectors that do not have a medical background. Data collector profile and training will vary by country depending on local requirements.

Finally, we aim to triangulate self-reported information with data extracted from MNH cards which will be reviewed at baseline and endline. The data included in these cards vary by country (supplemental materials). In India, Kenya, and South Africa the MNH cards are patient held. In Ethiopia, the maternal health card is based at the health facility where she receives ANC. Permissions to look at the maternal health records will be obtained from respondents.

Respondents will be compensated for their participation. At recruitment, women will be offered a mobile phone (to ensure they can do the phone surveys). Those who already own a phone will be offered the equivalent monetary value in airtime. Respondents will also receive airtime after completion of each monthly phone survey as well as compensation for participating in the in-person endline survey (e.g. a changing pad and diapers). They will also be reimbursed any transport cost to attend the end line survey.

### Analysis plan

The MNH eCohort aims to answer novel questions to guide health system improvements and to fill data gaps in implementing countries. For example, the second Ethiopian Health Sector Transformation Plan called for improvements in compassionate and respectful care [39]. Yet, little data exist on the level of respectful, user-centered care in the country. In South Africa, improving care quality for pregnant teenagers and HIV positive women is a priority.

Sample research questions across five key subdomains of the HQSS framework are shown in Table 3. The data generated could be used for a number of purposes including for monitoring quality, for benchmarking across sub-national regions, to guide clinical education reforms, direct the allocation of resources, or to standardize quality measurement [34].

**Table 3.** Novel research and policy-relevant questions.

Research questions		Key variables included in analyses
**Competent care**		
	What proportion of women and newborns receive a minimum set of recommended items in a timely manner during the antenatal and postnatal periods?	Content of antenatal care Gestational age at each ANC visit Content of postnatal care Child age at each PNC visit
**Competent systems**		
	Are women given the appropriate risk classification during ANC given pre-pregnancy history, and do they receive care that is appropriate for this classification?	Risk profile at first ANC visit (age, multiple pregnancy, concurrent chronic illness, prior obstetric complications, danger signs) Care content and care intensity for women with risk factors. Receiving ANC in hospital or with an obstetrician or specialist.
	Are emerging health problems detected, communicated, and treated in a timely manner over the course of care?	Danger signs and health problems reported during pregnancy and the postpartum period Testing and screening performed during ANC and PNC Women’s knowledge of their test results
	How many facilities do women visit over the continuum of care?	Number and type of facilities used for ANC, delivery and PNC (public, private, primary, secondary)
**Positive user experience**		
	Is user experience associated with changes in facility selection over the course of care?	User ratings of overall quality during the first and subsequent ANC visits User ratings of provider knowledge, respect, wait times, clarity of explanations, visit length, availability of equipment and medicine, courtesy of staff Facilities (number and types) used for ANC, delivery and PNC
	Is a positive user experience linked to better patient-reported outcomes?	User ratings of overall quality during the first and subsequent ANC visits Respectful care and mistreatment during childbirth Self-reported health during pregnancy and after delivery Symptoms of depression Experience of pregnancy and postpartum danger signs Infant Health-related quality of life
**Health outcomes**		
	Adjusting for socioeconomic differences among respondents, is higher quality care associated with higher self-rated health and health-related quality of life?	Education, wealth, employment, marital status, income Quality of ANC and neonatal outcomes Self-reported health during pregnancy and after delivery Symptoms of depression Experience of pregnancy and postpartum danger signs Infant Health-related quality of life
	What proportion of women receive minimally adequate treatment for postpartum depression?	Symptoms of depression Receipt of psychological counselling Number and length of counseling sessions Use of antidepressant medication
**Confidence in the health system**		
	Is higher quality care associated with improvements in health system trust over the course of care?	Quality of ANC and PNC Trust and endorsement in the health system
	What proportion of women would recommend their delivery care provider to a family member or friend?	Facility endorsement over the course of care and for delivery
**Economic outcomes**		
	How often do women forgo care (including visits, diagnostic tests, or medicine) because of cost?	Reasons for not seeking care during pregnancy, delivery and postpartum
	How many women employ coping mechanisms to pay for delivery care?	Income Costs of care Financial sources used to pay for care

## Discussion

To our knowledge, few studies have aimed to assess longitudinal MNH care quality in LMICs. Several health facilities surveys assess process quality for MNH care including the Service Provision Assessment (SPA), the World Bank Service Delivery Indicators survey and the WHO Harmonized Health Facility Assessment (HHFA) tool [4,40,41]. These surveys employ observations of ANC consultation and delivery care, health provider vignettes, health record reviews, and patient exit interviews. However, there is no longitudinal follow up of patients. Although pregnancy and childbirth require continuous care and multiple contacts with the health system, most surveys assessing MNH care quality are cross-sectional and do not follow women after they leave the health facility [42]. They are also largely focused on the facility and health providers rather than on the patients or users themselves.

Population-based surveys, including the DHS and the MICS, have also assessed the quality of maternal and newborn care [9,10]. In these surveys, women are asked to recall specific care items (e.g. blood pressure monitoring, and urine and blood testing) conducted during pregnancy for their last-born child in the last two or three years. They provide information on whether these items were done at least once in previous pregnancies, but not how many times and when. The PMA project has implemented a panel survey in Ethiopia which followed pregnant women at 6 weeks, 6 months, and one year postpartum [8]. The surveys are conducted face-to-face by resident enumerators and aim to measure the comprehensiveness of reproductive and MNH care, and the barriers and facilitators to such care.

The MNH eCohort differs from these existing tools. The eCohort will provide a detailed longitudinal picture of women’s experience over the course of care from first ANC visit to 10–12 weeks postpartum. It will focus on processes of care and outcomes from the woman’s perspective [7]. The eCohort will provide information on undermeasured dimensions of health system competence including continuity, integration, timeliness, and the ability of health systems to risk stratify and to prevent and detect health problems. Finally, the eCohort will combine the maternal and neonatal experience which are often treated separately in existing studies. In the future, the eCohort tool could be used as a follow-up module to expand patient exit interviews such as those conducted with ANC patients in SPA surveys. Women whose ANC consultation was observed in SPA surveys could be followed up until delivery through mobile phone surveys to assess longitudinal care quality and combine process measures and outcomes.

Nonetheless, the eCohort faces several limitations. First, unlike the SPA, DHS or MICS surveys, our study is not currently designed to provide nationally representative estimates but rather we aim for results to be representative of the health care system in the two sentinel sites selected in each country. Second, data on content of care items such as for specific laboratory tests or procedures will be limited by women’s recall. Studies have found varying levels of validity from user-reported care items [24,25,43,44]. However, unlike in several previous studies, women will be asked to recall the content of visits that have taken place recently (either on the same day or within the previous four weeks). We therefore expect validity to be higher than with longer recall periods. In future adaptations, competent care items could be extracted from EHRs in settings where EHRs are available. We also aim to complement these self-reported data, with data extracted from maternal and newborn health cards. However, the MNH cards reviewed may be incomplete or entirely missing, especially at endline. The limitations of paper-based maternal health records have been reported previously [25]. In cases where the card review is not possible, the assessment of care quality will be limited to women’s reports. Beyond recall issues, self-reported information may also be limited by social desirability bias where women may report better health behaviors or better-quality care than that received. However, some have shown that phone surveys perform better in avoiding social desirability bias compared to in-person surveys [45,46]. Finally, the measurement itself may bias results by informing women about the services they should be receiving, increase their expectations, and potentially motivating them to seek or demand additional care. The eCohort may therefore overestimate quality. Nonetheless, given poor performance in the study settings, the eCohort study is unlikely to substantially increase care quality for respondents. Similarly, the provision of phones could affect women’s experience at baseline. Finally, losses to follow-up could potentially exclude worst-off women or women with more serious health problems.

Improving health system quality measurement is crucial to designing policies for improved health system performance. The MNH eCohort aims to contribute to these efforts by testing a novel tool centered around competent systems and positive experiences and outcomes for mothers and newborns. Previous studies have demonstrated the feasibility of using mobile phone surveys to follow pregnant women in low-resource settings [47,48]. Nonetheless, implementation of the mobile phone MNH eCohort could face a myriad of challenges including with phone ownership and distribution, retention in the cohort, and tracing of respondents. Implementation components, survey costs and lessons learned from pilot testing in Ethiopia, Kenya, India, and South Africa will be described in a following paper. We also aim to make the final full survey instrument publicly available after pre-testing. In the future, principles of the eCohort could be applied to other health conditions that require longitudinal care and multiple contacts with the health system. For example, quality of care for chronic conditions such as hypertension, diabetes, cancer, or mental health could be monitored using an eCohort approach.

## Supplementary Material

Supplemental materials MNH eCohort Jul 21.docx
