# Supplementary material for: The maternal and newborn health eCohort to track longitudinal care quality: study protocol and survey development
Source: Glob Health Action. 2024 Aug 20;17(1):2392352. doi: 10.1080/16549716.2024.2392352 (PMC11338195; doi:10.1080/16549716.2024.2392352)
Supplement: Supplemental materials MNH eCohort Jul 21.docx [file ZGHA_A_2392352_SM1644.docx]

**Supplemental materials**

**The maternal and newborn health eCohort to track longitudinal care quality: study protocol and survey development**

[eCohort instrument sections by module 2](#_Toc172467140)

[eCohort sample size justification 7](#_Toc172467141)

[eCohort follow-up time 8](#_Toc172467142)

[Maternal health cards identified in each country 9](#_Toc172467143)

# eCohort instrument sections by module

**MODULE 0: RAPID HEALTH FACILITY ASSESSMENT**

A. HEALTH FACILITY META DATA

1. STAFFING

2. BASIC AMENITIES

3. AVAILABLE SERVICES

4. BASIC EQUIPMENT AND DIAGNOSTICS

5. REFERRAL SYSTEMS

6. PAYMENT FOR SERVICES

7. RECORD KEEPING AND HMIS

8. FACILITY CASE SUMMARY

**MODULE 1: BASELINE IN-PERSON ENROLLMENT SURVEY**

**A.** META DATA

**B.** RESPONDENT ELIGIBILITY

1. RESPONDENT IDENTIFICATION
2. HEALTH PROFILE
   1. SELF-RATED HEALTH
   2. CHRONIC CONDITIONS AND MEDICATION
   3. FUNCTION (EQ5D)
   4. DEPRESSION (PHQ9)
3. OVERALL RATINGS OF THE HEALTH SYSTEM AND PATIENT ACTIVATION
4. CARE PATHWAYS
   1. TRANSPORT AND TRAVEL TIME
   2. REASONS FOR CHOOSING CURRENT FACILITY
5. BASIC DEMOGRAPHICS
   1. SOCIAL SUPPORT
   2. HEALTH BELIEFS/KNOWLEDGE
   3. PHONE AND ADDRESS
6. VISIT TODAY: USER EXPERIENCE
7. VISIT TODAY: CONTENT OF CARE
   1. EXAMINATIONS AND INVESTIGATION
   2. TREATMENTS
   3. COUNSELING AND SCREENING
   4. INSTRUCTIONS AND ADVANCED CARE
8. CURRENT PREGNANCY
   1. EXPECTED DUE DATE AND DELIVERY PLAN
   2. COMMON PROBLEMS IN PREGNANCY
   3. SIGNS OF EMERGENCY (RED FLAGS) AND MANAGEMENT DURING VISIT
9. RISKY HEALTH BEHAVIORS
10. OBSTETRIC HISTORY
    1. PARITY, GRAVIDITY, AND NEONATAL DEATHS
11. INTIMATE PARTNER VIOLENCE
12. ECONOMIC STATUS AND OUTCOMES
    1. ASSETS
    2. COST OF VISIT
    3. INSURANCE
    4. CLOSING QUESTION: SATISFACTION
13. WOMAN PHYSICAL ASSESSMENTS
    1. HEIGHT
    2. WEIGHT
    3. BLOOD PRESSURE
    4. ANEMIA TEST
14. SURVEY CONCLUSION

**MODULE 2: REPEATED PHONE SURVEYS IN THE PRENATAL PERIOD**

A. INTRODUCTION

1. IDENTIFICATION
2. GENERAL HEALTH (ALL WOMEN)
   1. SELF-REPORTED HEALTH
   2. SIGNS OF EMERGENCY
   3. DEPRESSION
   4. SMOKING
3. CARE PATHWAYS (ALL WOMEN)
   1. NUMBER OF NEW VISITS
   2. LOCATION OF NEW VISIT(S)
   3. MAIN PURPOSE OF NEW VISIT(S)
4. USER EXPERIENCE IN EACH VISIT (WOMEN WITH NEW VISITS)
   1. UX 1ST NEW CONSULTATION
   2. UX 2ND NEW CONSULTATION
   3. UX 3RD NEW CONSULTATION
   4. UX 4TH NEW CONSULTATION
   5. UX 5TH NEW CONSULTATION
5. CONTENT OF CARE SINCE LAST INTERVIEW (WOMEN WITH NEW VISITS)
   1. NEW DIAGNOSTIC TESTS
   2. RECEIPT OF TEST RESULTS
   3. NEW COUNSELLING
   4. MANAGEMENT OF SIGNS OF EMERGENCY
   5. MANAGEMENT OF DEPRESSION
   6. NEW INSTRUCTIONS
6. NEW MEDICATIONS OR SUPPLEMENTS (ALL WOMEN)
7. COSTS (NEW VISITS)

B. CONCLUSION

**MODULE 3: FIRST PHONE SURVEY AFTER CHILDBIRTH OR END OF PREGNANCY**

1. PREGNANCY OUTCOME AND NEWBORN HEALTH
   1. SIZE AND WEIGHT
   2. REPORTED HEALTH
   3. INFANT FEEDING
   4. INFANT HRQOL
   5. FETAL DEATH, STILLBIRTH, OR NEWBORN DEATH
2. ADDITIONAL ANTENATAL CARE
   1. MAIN PURPOSE OF NEW VISIT(S)
   2. NEW DIAGNOSTIC TESTS
3. CARE PATHWAYS DURING LABOR AND DELIVERY
   1. FIRST FACILITY VISITED
   2. DELIVERY FACILITY
4. CONTENT OF INTRAPARTUM CARE
   1. LABOR AND DELIVERY CARE
   2. C-SECTION AND EPISIOTOMY
   3. IMMEDIATE POSTPARTUM CARE
   4. DISCHARGE AND COUNSELLING
   5. RECORDS
   6. CARE FOR HOME BIRTHS 23
   7. PNC CARE PLAN
5. MATERNAL AND NEWBORN COMPLICATIONS
   1. MATERNAL COMPLICATIONS
   2. NEONATAL COMPLICATIONS OR DANGER SIGNS
6. MATERNAL POST PARTUM HEALTH
   1. DEPRESSION (PHQ2)
   2. MANAGEMENT OF DEPRESSION
   3. POSTPARTUM DANGER SIGNS
   4. OBSTETRIC FISTULA
7. NEW MEDICATIONS OR SUPPLEMENTS
   1. MEDICATION OR SUPPLEMENT FOR THE WOMAN
   2. MEDICATION OR SUPPLEMENT FOR THE BABY
8. USER EXPERIENCE
   1. MISTREATMENT DURING CHILDBIRTH
   2. CONSENT AND PRIVACY FOR VAGINAL EXAMS
   3. APPROPRIATE PAIN RELIEF
9. ECONOMIC OUTCOMES

B. CONCLUSION

1. MISCARRIAGE/ABORTION SURVEY CONCLUSION

**MODULE 4: PHONE FOLLOW UP AFTER CHILDBIRTH (6-8 WEEKS POSTPARTUM)**

A. INTRODUCTION

1. IDENTIFICATION
2. HEALTH – BABY
   1. REPORTED HEALTH
   2. INFANT FEEDING
   3. INFANT HRQOL
   4. INFANT DANGER SIGNS
   5. NEWBORN/INFANT DEATH
3. HEALTH – WOMAN
   1. SELF-RATED HEALTH
   2. DEPRESSION (PHQ2)
   3. MOOD AND ATTACHMENT
   4. PAIN WITH INTERCOURSE
   5. OBSTETRIC FISTULA
4. CARE PATHWAYS
   1. NUMBER OF NEW VISITS
   2. LOCATION OF NEW VISIT(S)
   3. MAIN PURPOSE OF NEW VISIT(S)
   4. TIMING OF NEW VISIT(S)
5. USER EXPERIENCE IN EACH VISIT
   1. UX 1ST NEW CONSULTATION
   2. UX 2ND NEW CONSULTATION
   3. UX 3RD NEW CONSULTATION
6. CONTENT OF CARE SINCE DELIVERY – BABY
   1. EXAMINATIONS AND TESTS
   2. COUNSELLING AND SCREENING
   3. MANAGEMENT OF SIGNS OF EMERGENCY
7. CONTENT OF CARE SINCE DELIVERY – WOMAN
   1. EXAMINATIONS, TESTS
   2. POST C-SECTION CARE
   3. COUNSELLING AND SCREENING
   4. MANAGEMENT OF DEPRESSION
8. NEW MEDICATIONS, VACCINES OR SUPPLEMENTS
   1. MEDICATION OR SUPPLEMENT FOR THE WOMAN
   2. MEDICATION OR SUPPLEMENT FOR THE BABY
   3. VACCINES FOR THE BABY
9. COST OF VISITS

B. SURVEY CONCLUSION

**MODULE 5: ENDLINE IN-PERSON SURVEY (10-12 WEEKSPOSTPARTUM)**

A. INTRODUCTION

1. IDENTIFICATION
2. HEALTH – BABY
   1. R EPORTED HEALTH
   2. INFANT FEEDING
   3. INFANT HRQOL
   4. INFANT DANGER SIGNS
   5. INFANT DEATH
3. HEALTH – WOMAN
   1. SELF-RATED HEALTH
   2. FUNCTION (EQ5D)
   3. DEPRESSION (PHQ2)
   4. MOOD AND ATTACHMENT
   5. PAIN WITH INTERCOURSE
   6. OBSTETRIC FISTULA
4. OVERALL RATINGS OF THE HEALTH SYSTEM AND PATIENT ACTIVATION
   1. HEALTH SYSTEM CONFIDENCE
   2. PATIENT ACTIVATION
   3. MEDICAL ERROR AND DISCRIMINATION
5. CARE PATHWAYS
   1. NUMBER OF NEW VISITS
   2. LOCATION OF NEW VISIT(S)
   3. MAIN PURPOSE OF NEW VISIT(S)
6. USER EXPERIENCE IN EACH VISIT
   1. UX 1ST NEW CONSULTATION
   2. UX 2ND NEW CONSULTATION
   3. UX 3RD NEW CONSULTATION
7. CONTENT OF CARE SINCE LAST INTERVIEW– BABY
   1. EXAMINATIONS AND TESTS
   2. COUNSELLING AND SCREENING
   3. MANAGEMENT OF SIGNS OF EMERGENCY
8. CONTENT OF CARE SINCE LAST INTERVIEW – WOMAN
   1. EXAMINATIONS, TESTS
   2. POST C-SECTION CARE
   3. COUNSELLING AND SCREENING
   4. MANAGEMENT OF DEPRESSION
9. NEW MEDICATIONS, VACCINES OR SUPPLEMENTS
   1. MEDICATION OR SUPPLEMENT FOR THE WOMAN
   2. MEDICATION OR SUPPLEMENT FOR THE BABY
   3. VACCINES FOR THE BABY
10. COST OF VISITS
11. INTIMATE PARTNER VIOLENCE
12. CLOSING QUESTION: SATISFACTION
13. WOMAN PHYSICAL ASSESSMENTS
    1. HEIGHT
    2. WEIGHT
    3. BLOOD PRESSURE
    4. ANEMIA TEST
14. BABY PHYSICAL ASSESSMENTS
    1. WEIGHT
    2. LENGTH
    3. HEAD CIRCUMFERENCE

B. SURVEY CONCLUSION

# eCohort sample size justification

We estimate that a minimum of 385 women per site will be needed to obtain estimates that are representative of each of the selected sites. The sample size was determined by using single population proportion formula as follows.

$$n=\frac{Z{(\alpha/2)}^{2} *p(1-p)}{{(SE)}^{2}}$$

where n is the sample size, Z is the test statistic for the normal distribution at 95% confidence interval for two-sided test i.e. 1.96, α is type I error (5%), p is the proportion of women with the outcome variable (for example, the proportion of women who rate the quality of their first antenatal care visit as good or excellent) and SE is the standard error of sampling (5%). As the prevalence of the outcomes of interest is not known, we used a 50% prevalence which maximizes the sample size. Substituting the above numbers in the formula above gives a sample size of 384.2 women.


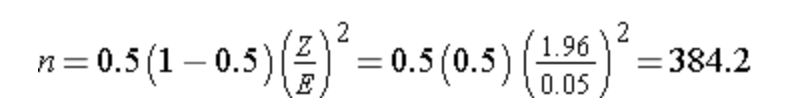


Similar longitudinal studies, such as the PMA 2020 study, have reported losses to follow-up rates ranging from 16% to 22%. The PMA 2020 study only included three rounds of follow up. Given that we estimate a median of 6 follow-ups per woman, we decided to account for a potential maximum loss to follow up of 30%. This led to our decision to recruit a total of 500 women in each site (385+30%).

**References:**

**PMA 2020:**

[https://www.pmadata.org/sites/default/files/data_product_results/Kenya Kitui_Phase 3_Panel_Results Brief_Final.pdf](https://www.pmadata.org/sites/default/files/data_product_results/Kenya%20Kitui_Phase%203_Panel_Results%20Brief_Final.pdf)

<https://www.pmadata.org/sites/default/files/data_product_results/KEP3_Kilifi_Panel%20Results%20Brief_Draft%206.pdf>

# eCohort follow-up time

The follow-up time will vary based on gestational age at enrollment. Ideally, women will attend their first antenatal care visit in the first trimester. However, in the implementing countries, it is not uncommon for women to seek ANC for the first time in the second trimester or even third. The figure below describes the range in potential follow-up times for eCohort respondents.

Longest potential follow-up time (woman seeks her first ANC visit and is enrolled in eCohort early in pregnancy at 4-6 weeks’ gestation):

|  | Prenatal period | | | | | | | | | Postnatal period | | |
| --- | --- | --- | --- | --- | --- | --- | --- | --- | --- | --- | --- | --- |
| Module | M1 | M2 | M2 | M2 | M2 | M2 | M2 | M2 | M2 | M3 | M4 | M5 |
| GA at enrollment | 4-6 weeks | 8-10 | 12-14 | 16-18 | 20-22 | 24-26 | 28-30 | 32-34 | 36-38 | + 2-4 weeks | + 6-8 weeks | + 10-12 weeks |
| Time elapsed in months | 0 | 1 | 2 | 3 | 4 | 5 | 6 | 7 | 8 | 9 | 10 | **11 months** |

Shortest potential follow-up time (woman seeks her first ANC visit and is enrolled in eCohort late in pregnancy, at 36-38 weeks’ gestation):

|  | Prenatal period | Postnatal period | | |
| --- | --- | --- | --- | --- |
| Module | M1 | M3 | M4 | M5 |
| GA at enrollment | 36-38 weeks | + 2-4 weeks | + 6-8 weeks | + 10-12 weeks |
| Time elapsed in months | 0 | 1 | 2 | **3 months** |

# Maternal health cards identified in each country

|  | **Type of card/record** |
| --- | --- |
| **Ethiopia** | Integrated Antenatal, Labor, Delivery, Newborn and Postnatal Care Card (Federal Ministry of Health)  Available from: *MANAGEMENT PROTOCOL ON SELECTED OBSTETRICS TOPICS. Federal Democratic Republic of Ethiopia, Ministry of Health, January 2010.* |
| **India** | MINISTRY OF HEALTH AND FAMILY WELFARE, MINISTRY OF WOMEN AND CHILD DEVELOPMENT, MOTHER AND CHILD PROTECTION CARD  Available from: [*https://nhm.gov.in/New_Updates_2018/NHM_Components/Immunization/Guildelines_for_immunization/MCP_Card_English_version.pdf*](https://nhm.gov.in/New_Updates_2018/NHM_Components/Immunization/Guildelines_for_immunization/MCP_Card_English_version.pdf) |
| **Kenya** | Mother and child handbook. Revised Edition September 2020  Available from*:* [*https://familyhealth.go.ke/wp-content/uploads/2020/11/Mother-Child-Health-Handbook-MOH-September-2020.pdf*](https://familyhealth.go.ke/wp-content/uploads/2020/11/Mother-Child-Health-Handbook-MOH-September-2020.pdf) |
| **South Africa** | Maternity case record, Department of Health.  Described in: *Guidelines for Maternity care in South Africa. A manual for clinics, community health centers and district hospitals. Fourth edition 2016. Department of Health. Republic of South Africa.* [*https://knowledgehub.health.gov.za/system/files/elibdownloads/2023-04/CompleteMaternalBook.pdf*](https://knowledgehub.health.gov.za/system/files/elibdownloads/2023-04/CompleteMaternalBook.pdf) |
